# Differentiation of Brain Abscess From Cystic Glioma Using Conventional MRI Based on Deep Transfer Learning Features and Hand-Crafted Radiomics Features

**DOI:** 10.3389/fmed.2021.748144

**Published:** 2021-11-12

**Authors:** Linlin Bo, Zijian Zhang, Zekun Jiang, Chao Yang, Pu Huang, Tingyin Chen, Yifan Wang, Gang Yu, Xiao Tan, Quan Cheng, Dengwang Li, Zhixiong Liu

**Affiliations:** ^1^Shandong Key Laboratory of Medical Physics and Image Processing, Shandong Institute of Industrial Technology for Health Sciences and Precision Medicine, School of Physics and Electronics, Shandong Normal University, Jinan, China; ^2^Department of Oncology, Xiangya Hospital, Central South University, Changsha, China; ^3^Department of Network Information Center, Xiangya Hospital, Centra South University, Changsha, China; ^4^Department of Neurosurgery, Xiangya Hospital, Central South University, Changsha, China; ^5^National Clinical Research Center for Geriatric Disorders, Xiangya Hospital, Central South University, Changsha, China

**Keywords:** brain abscess, deep transfer learning, radiomics, convolutional neural network, cystic glioma

## Abstract

**Objectives:** To develop and validate the model for distinguishing brain abscess from cystic glioma by combining deep transfer learning (DTL) features and hand-crafted radiomics (HCR) features in conventional T1-weighted imaging (T1WI) and T2-weighted imaging (T2WI).

**Methods:** This single-center retrospective analysis involved 188 patients with pathologically proven brain abscess (102) or cystic glioma (86). One thousand DTL and 105 HCR features were extracted from the T1WI and T2WI of the patients. Three feature selection methods and four classifiers, such as k-nearest neighbors (KNN), random forest classifier (RFC), logistic regression (LR), and support vector machine (SVM), for distinguishing brain abscess from cystic glioma were compared. The best feature combination and classifier were chosen according to the quantitative metrics including area under the curve (AUC), Youden Index, and accuracy.

**Results:** In most cases, deep learning-based radiomics (DLR) features, i.e., DTL features combined with HCR features, contributed to a higher accuracy than HCR and DTL features alone for distinguishing brain abscesses from cystic gliomas. The AUC values of the model established, based on the DLR features in T2WI, were 0.86 (95% CI: 0.81, 0.91) in the training cohort and 0.85 (95% CI: 0.75, 0.95) in the test cohort, respectively.

**Conclusions:** The model established with the DLR features can distinguish brain abscess from cystic glioma efficiently, providing a useful, inexpensive, convenient, and non-invasive method for differential diagnosis. This is the first time that conventional MRI radiomics is applied to identify these diseases. Also, the combination of HCR and DTL features can lead to get impressive performance.

## Introduction

Brain glioma is the most common intracranial brain tumor that is extremely difficult to treat. Currently, surgical resection is the standard treatment of resectable diseases, followed by postoperative radiotherapy and chemotherapy (1). The majority of gliomas are solid tumors, but some present cystic changes, such as cystic glioma, which has different clinicopathological features from other tumors. Brain abscess is an infectious disease that has high morbidity and mortality (2, 3). Though the treatment and prognosis of these two diseases are different, accurate and timely differential diagnosis is crucial. In many cases, CT and MR images lack specificity for cystic glioma and brain abscess, especially when the medical history and clinical manifestations of the diseases cannot provide a differential diagnosis for timely treatment measures. At present, the two diseases are mainly distinguished by pathological examination, with the caveat of invasive procedure and intra-operator variability. To accurately distinguish the two diseases, previous studies have proposed advanced MR images diagnosis techniques (2, 4), such as susceptibility-weighted imaging and apparent diffusion coefficients (ADC). However, these diagnosis techniques cannot obtain high accuracy, and they rely on the experience of radiologists (5). The use of the most rudimentary imaging modalities of T1-weighted imaging (T1WI) and T2-weighted imaging (T2WI) for a training model with a large sample size contributes to more universality and fewer errors.

As a method of machine learning, radiomics is used for quantitative image feature extraction from tumor regions of interest. It has great potential for oncology practice, including differential diagnosis, prediction of pathological classification, lymph node metastasis, and survival (6–10). Radiomics has been applied to brain tumor diseases (11–15), especially in differentiating brain tumors (16–21). For example, Qian et al. investigated the ability of radiomic analysis to distinguish between isolated brain metastases and glioblastoma (16); Dong et al. used the radiomic features derived from the areas of peripheral enhancing edema to differentiate glioblastoma from supratentorial single brain metastasis (17); Zhang et al. investigated the feasibility of contrast-enhanced T1WI radiomics features extracted by machine-learning algorithms to distinguish between low-grade oligodendroglioma and atypical anaplastic oligodendroglioma (18); Chen et al. applied radiomics analysis to distinguish between metastatic brain tumors and glioblastomas based on contrast-enhanced T1WI, and they validated the discriminative performance of this method (19); Artzi et al. used radiomics-based machine learning to differentiate between brain metastasis subtypes and glioblastoma based on conventional postcontrast T1WI (20). However, the radiomics features are mainly the texture, size, volume, shape, and intensity characteristics of the tumor, limiting the potential of this method. Therefore, extracting more complex features and fusing them with radiomic features may improve the prediction and generalization capabilities of the model (21–23).

In recent years, deep convolutional neural networks (CNNs) (24) with complex network structures have achieved remarkable results in the field of computer vision, such as tumor grade prediction, patient prognosis, pathology classification, and organ segmentation (25, 26). The successful application of deep learning requires a large number of training cohort sets. Since the available medical data sets have a limited size, a pretrained CNN known as “transfer learning” can be employed to avoid overfitting and replace deep learning in many practical applications (21, 27, 28).

It is not clear whether T1WI and T2WI, as conventional routine images in hospitals, also have diagnostic values for distinguishing brain abscess from cystic glioma. In this study, we hypothesized that conventional T1WI and T2WI would also be valuable in distinguishing between these two diseases. To this end, the DLR features extracted from patients with brain abscesses or cystic gliomas were used to validate the diagnostic capability of T1WI and T2WI.

## Materials and Methods

### Patients

This study was reviewed and approved by the Institutional Review Board of Xiangya Hospital and informed consent was provided by the patient participating in this study. From January 2017 to October 2020, 216 patients who met requirements and underwent T1WI and T2WI MRI were included in the cohort after an initial case screening. Twenty-eight patients were excluded due to poor MRI quality caused by technical operations or inspection processes. Finally, 188 patients were enrolled in this study, among which, 102 patients were diagnosed with brain abscesses (age [mean ± SD], 47.8 ± 17.6 years; 33 males and 69 females) and 86 were diagnosed with cystic gliomas (age [mean ± SD], 46.2 ± 15.1 years; 27 males and 59 females). The training cohort and test cohort were divided by stratified sampling with a ratio of 7:3, and the distribution of the two diseases was almost the same as that of the overall data set. Then, a nested 5-fold cross-validation was performed on the training cohort.

**Figure 1:**
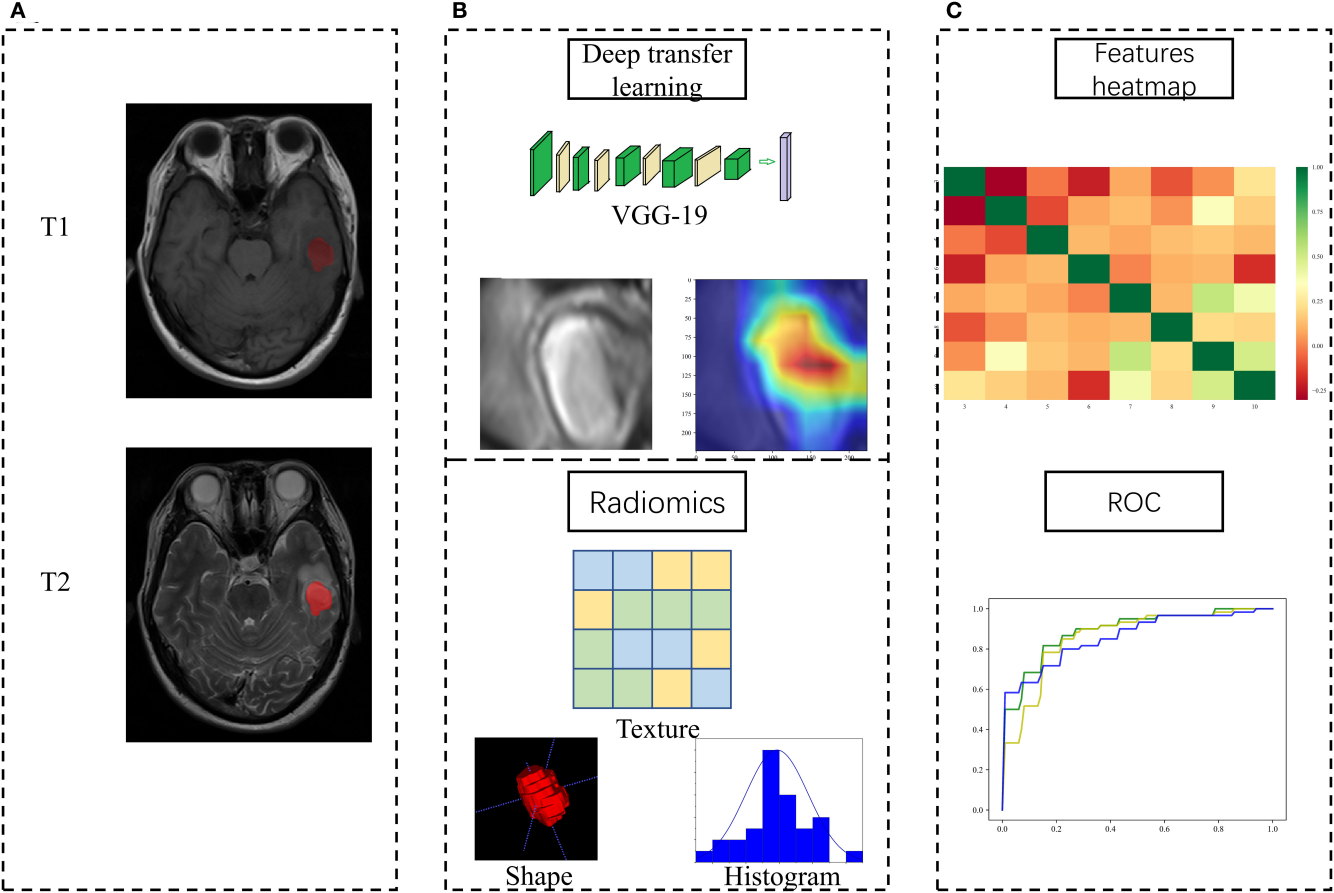
Study workflow overview. **(A)** Imaging processing; **(B)** Feature extraction; **(C)** Feature analysis and model construction.

Figure 1 showed the flowchart of our study, consisting of image preprocessing, feature extraction, feature analysis, and model construction.

### Image Acquisition

All MRI examinations were conducted in the radiology department of Xiangya hospital with a 3.0T MR Scanner. High-quality MR images were obtained under the following configurations: ①axial T1WI: layer thickness = 5 mm, layer spacing = 1.5 mm, matrix = 512 × 416, field of view = 24 × 24 cm. ②axial T2WI: layer thickness = 5 mm, layer spacing = 1.5 mm, matrix = 416 × 512, field of view = 24 × 24 cm. All MR images were retrieved from the picture archiving and communication system for further image feature extraction.

### Image Preprocessing and Tumor Segmentation

To convert T1WI images to the space of T2WI images, automatic rigid registration was performed with the ITK-SNAP software (Version 3.8.0, http://www.itksnap.org/) to segment the structures in the 3D medical image. Meanwhile, manual segmentation of the lesions of all subjects was performed on registered T2WI and T1WI images by a neuroradiologist with 10 years of experience. Then, a radiologist (with 10-years experience) segmented 50 cases, consisting of 25 pathologically proven brain abscess cases and 25 cystic glioma cases randomly selected from all samples. In this way, the consistency of the extracted HCR and DTL features of the neuroradiologist and radiologist was evaluated, and the impact of inter-operator variation on model stability and generalizability was reduced. Besides, the intra-class correlation coefficient of each feature of these 50 cases is calculated.

Image preprocessing was performed as follows. Before DTL features were extracted, an image of the largest cross-sectional area and its upper and lower layers were chosen as a three-channel image. Then, a rectangular region of interest around the tumor contour was used to crop the MR image. Next, the size of the tumor patch was resized to 224 × 224 to meet the input size requirement of the pretrained CNN model. Before the HCR features were extracted, B-spline interpolation was adopted to resample all images to the same voxel size of 1 × 1 × 1 mm^3^. To avoid the influence of different MR image machine scanners on feature extraction, all images were normalized. Moreover, it seems that deep learning is less affected by different MR machine types than radiomics.

### Feature Extraction

In line with the Imaging Biomarker Standardization Initiative, two kinds of features were extracted, i.e., DTL feature and HCR feature. As for the extraction of the DTL feature, ResNet-50 (29) and VGG-19 (30) pretrained on the natural image dataset ImageNet (http://www.image-net.org/) were taken as our base models. (Visual Geometry Group) VGG-19 contains 19 hidden layers (16 convolution layers and 3 full connection layers). It uses 3 × 3 convolutional kernels in all layers to deepen the number of layers and avoid excessive parameters. As for ResNet, it integrates residual learning to avoid gradient dispersion and accuracy reduction in deep networks, improving the network efficiency, accuracy, and execution speed. The internal deep learning features in the image are also visualized while the convolutional layer receives the input features and generates the output feature mapping. As for the extraction of the HCR feature, 105 original HCR features were extracted from each of the axial T1WI and T2WI images using PyRadiomics (Version 2.1.0, https://pyradiomics.readthedocs.io/).

### Feature Selection

To prevent overfitting, the multistep feature selection method was adopted to select the best features for distinguishing brain abscesses from cystic gliomas. First, all the HCR features were analyzed in order by the Spearman rank correlation test and mutual information method. The Spearman rank correlation test was used to investigate the internal linear correlation between individual features. The higher the absolute value of the correlation coefficient, the stronger the correlation. As for the non-redundant features (with a linear correlation coefficient <0.95), the mutual information method was used to capture arbitrary relationships (both linear and non-linear) between each feature and object variable. Then, according to the imaging modality and feature category, the remaining radiomics features and all DLR features were divided into four feature groups, i.e., T1WI-HCR, T2WI-HCR, T1WI-DTL, and T2WI-DTL group. Finally, the least absolute shrinkage and selection operator (LASSO) and recursive feature elimination (RFE) method based on LR and SVM were adopted to repeatedly create the model and select the best feature subset in each feature group.

### Feature Fusion

**Figure 2:**
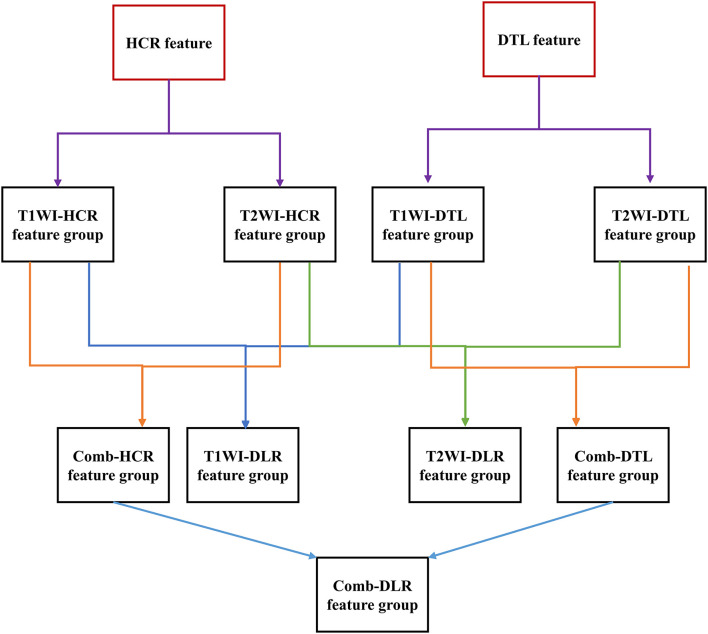
Feature selection and fusion flow chart. The red box is the original extracted feature. The black box is the feature group. Each feature group contains 10 features. A new feature group was formed after feature fusion and screening of two feature groups.

Feature fusion indicates that two feature groups are put together. The T1WI-HCR and T2WI-HCR feature groups were fused to combined HCR (comb-HCR), and 10 optimal MR image features were selected; similarly, T1WI-DTL and T2WI-DTL were fused to combined DTL (comb-DTL); the T1WI-HCR and T1WI-DTL feature groups were fused to T1WI-DLR, and 10 optimal MR image features were selected; the T2WI-HCR and T2WI-DTL feature groups were fused to T2WI-DLR, and 10 optimal MR image features were selected; the comb-HCR and comb-DTL feature groups were fused to combined DLR (comb-DLR), and 10 optimal MR image features were selected. Refer Figure 2 for details of feature selection and fusion flow chart.

### Feature Analysis/Model Construction and Validation

After feature fusion and selection, we used each feature group separately to build machine learning classification models, including LR, RFC, KNN, and SVM, implemented by Python Scikit-learn (https://scikit-learn.org/stable/user_guide.html). The performance of different classifiers was compared. To prevent overfitting, we performed 1,000 iterations of nested 5-fold cross-validation to select the best parameters for the classifier in the training cohorts. The discriminative power of the model was assessed by AUC values, Youden Index, and receiver operating characteristic (ROC) curves. Accuracy, precision, recall, specificity, and F1-score were also used as quantitative metrics. The AUC values for comparative disease identification were carried out using DeLong test.

### Clinical ADC Maps vs. Our Model

In this study, two experienced radiologists (with more than 10 years of experience in brain tumor MRI) were assigned to jointly perform image ADC diagnosis, but they were not involved in the quantitative image analysis described above. All clinicopathological information were removed, and the radiologists distinguished brain abscesses from cystic gliomas based on ADC images only. The proportion of ADC maps in our study cohort was counted. Besides, the diagnostic performance of the two radiologists on the same dataset following the current clinical practice (including the use of ADC maps) was compared to that of the established classifier.

### Statistical Analysis

The comparison of categorical variables was performed through chi-square tests or Fisher tests, and the comparison between quantitative variables was performed through *t*-tests or Mann-Whitney *U*-test. Meanwhile, the Spearman rank correlation test was adopted to evaluate the correlation and executed in Python. A *p* < 0.05 (two-sided) indicates a significant difference in distinguishing cystic gliomas from brain abscesses. Statistical analysis was performed with IBM SPSS Statistics (version 25; IBM Corporation, Armonk, NY, USA), R (https://cran.r-project.org/), and Python (version 3.6.6, https://www.python.org). A pretrained CNN model was run using Keras with a Tensorflow backend (https://keras.io/applications/#Resnet-50 and https://keras.io/applications/#VGG-19).

## Results

### Patient Characteristics

A total of 131 and 57 patients were involved in the training and test cohort of this study, respectively. Specifically, the training cohort involved 71 patients with brain abscesses and 60 patients with gliomas, while the test cohort involved 31 patients with brain abscesses and 26 patients with gliomas. The patient characteristics are provided in Supplementary Table 1. The gold standard for distinguishing between brain abscess and cystic glioma was confirmed by pathologists through pathological examination.

### Results of the Feature Extraction

To extract the DTL features, the tumor patch images were input to the pretrained CNN to extract 1,000 features from each MR image modality, and the extracted features were outputs from the last fully connected layer of VGG-19 and ResNet-50. The extracted HCR features included First Order Statistics (18 features), Shape-based (3D) (14 features), Gray Level Cooccurrence Matrix (22 features), Gray Level Run Length Matrix (16 features), Gray Level Size Zone Matrix (16 features), Neighboring Gray Tone Difference Matrix (5 features), and Gray Level Dependence Matrix (14 features).

### Results of the Feature Selection and Fusion

After feature selection and fusion, only the features with intra-class correlation coefficients >0.95 were retained, indicating that these features are not affected by multiple tumor segmentation operators and present high reproducibility. For single image modality analysis, a feature selection method was used to preserve 10 optimal features in each group. In multimodality analysis, the two groups of each modality were combined, and 10 features were filtered out. Refer Supplementary Table 2 for detailed feature selection results.

### DLR vs. DTL vs. HCR

**Figure 3:**
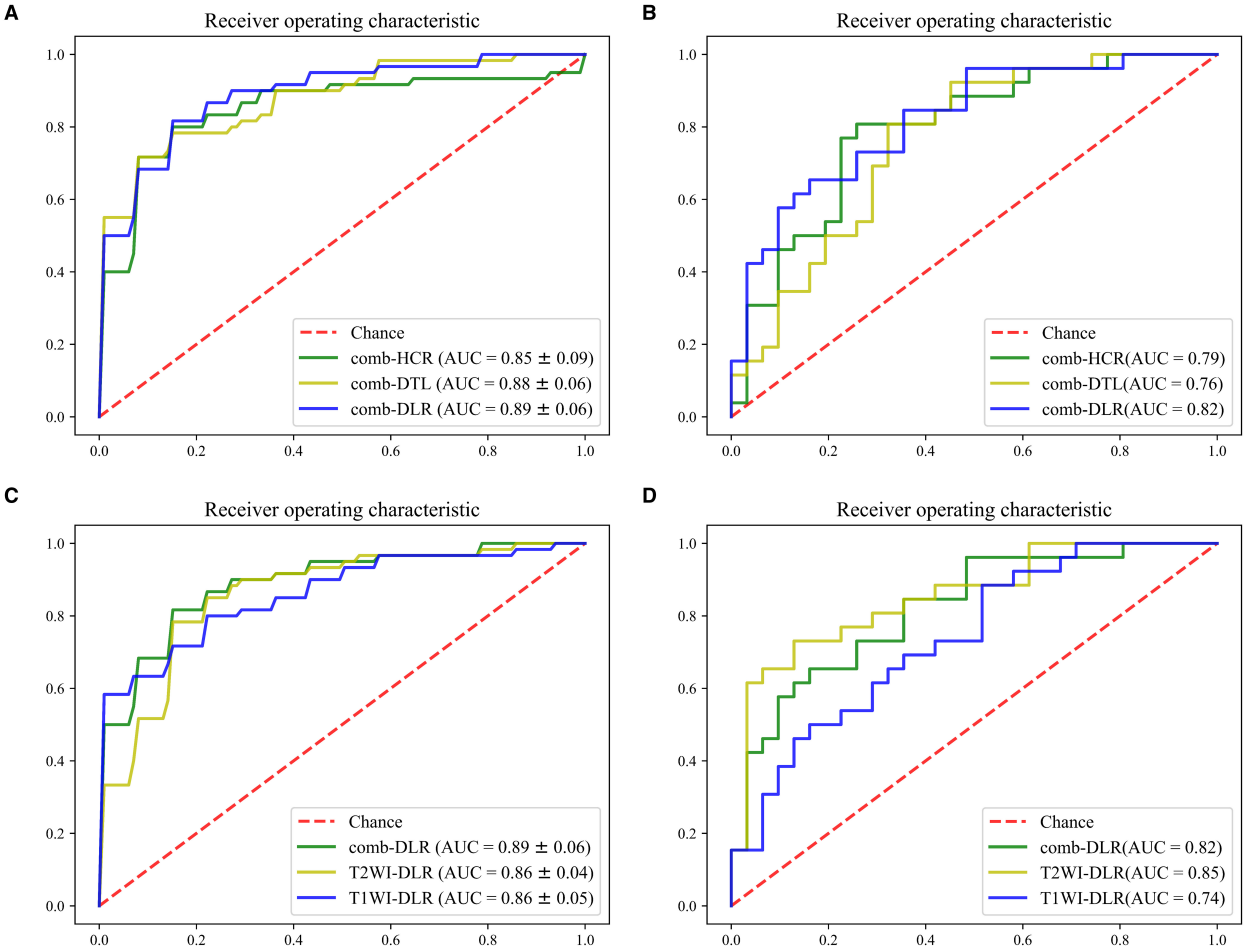
ROC comparison. **(A,B)**, comb-HCR vs. comb-DTL vs. comb-DLR in the training cohort **(A)** and test cohort **(B)**. **(C,D)** T1WI-DLR vs. T2WI-DLR vs. comb-DLR in the training cohort **(C)** and test cohort **(D)**.

The modeling effects of the combined modality feature with different category were compared. On the test cohort, the feature obtained from comb-DLR presented higher diagnostic accuracy than those from comb-HCR and comb-DTL for distinguishing brain abscess from cystic glioma. The AUC values of the models established with the comb-DLR, comb-HCR, and comb-DTL models features were 0.82, 0.79, and 0.76, respectively; the AUC values of the models established with the T2WI-DLR, T2WI-HCR, and T2WI-DTL features were 0.85, 0.80, and 0.71, respectively; the AUC values of the models established with the T1WI-DLR, T1WI-HCR, and T1WI-DTL features were 0.74, 0.77, and 0.80, respectively. Refer Figure 3, Table 1 for details of model comparison results.

**Table 1:** Model representation.

**Imaging modality** **and feature category**		**Features selection+** **Classifier**	**Training cohort(Cross validation)**	**Test cohort**					
			**Mean AUC** **(95% CI)**	**AUC** **(95% CI)**	**Accuracy**	**Precision**	**Recall**	**F1-score**	**Specificity**
HCR	T1 WI-HCR	RFE(LR)+LR	0.75 (95% CI: 0.61, 0.89)	0.79 (0.67–0.91)	0.67	0.65	0.77	0.61	0.74
	T2WI-HCR	RFE(LR)+SVM	0.84 (95% CI: 0.72, 0.96)	0.75 (0.62–0.88)	0.75	0.73	0.73	0.73	0.77
	comb-HCR	RFE(LR)+LR	0.85 (95% CI: 0.74, 0.96)	0.79 (0.68–0.91)	0.77	0.77	0.74	0.75	0.77
DTL	T1 WI-DTL	RFE(SVM)+SVM	0.89(95% CI: 0.84, 0.94)	0.81 (0.69–0.93)	0.74	0.76	0.62	0.68	0.84
	T2WI-DTL	RFE(SVM)+SVM	0.9(95% CI: 0.85, 0.95)	0.71 (0.58–0.85)	0.68	0.72	0.5	0.59	0.84
	comb-DTL	RFE(LR)+LR	0.88(95% CI: 0.81, 0.95)	0.77 (0.65–0.90)	0.65	0.69	0.42	0.52	0.84
DLR	T1 WI-DLR	RFE(LR)+LR	0.86(95% CI: 0.80, 0.92)	0.75 (0.63–0.88)	0.67	0.65	0.63	0.64	0.68
	T2 WI-DLR	RFE(SVM)+SVM	0.86(95% CI: 0.81, 0.91)	0.85 (0.75–0.95)	0.77	0.73	0.76	0.75	0.81
	comb-DLR	RFE(SVM)+SVM	0.89(95% CI: 0.82, 0.96)	0.83 (0.73–0.94)	0.72	0.65	0.71	0.68	0.77

### Multimodality vs. Single Modality

Since the AUC value of the model established with the DLR features was statistically higher than that of the models established with the HCR and DTL features in most cases, the DLR features were used in the multimodal experiments. On the test cohort, the AUC value of the model established with the T2WI-DLR features was statistically higher than that of the model established with the T1WI-DLR and com-DLR features. Model comparison results are provided in Figure 3, Table 1.

### Construction and Validation of the Final Model

**Figure 4:**
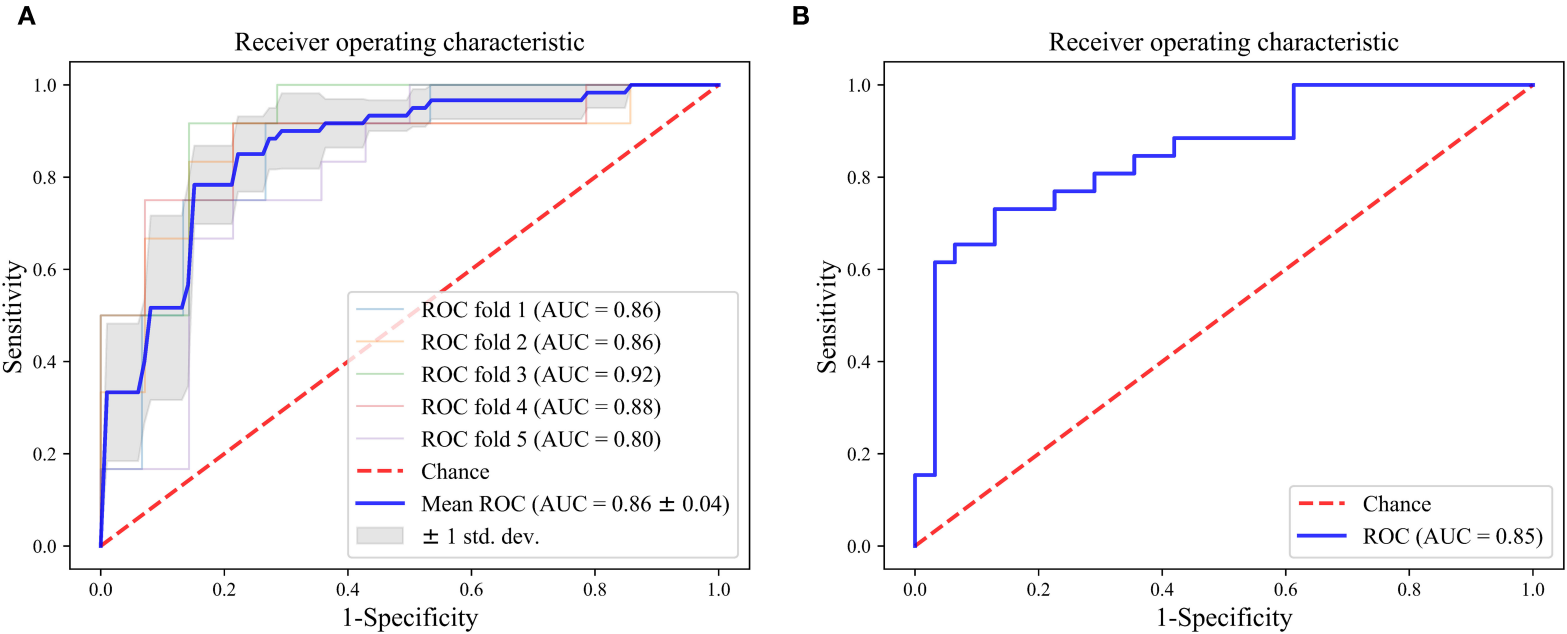
ROC curves of the optimal model (T2WI-DLR). **(A)** training cohort; **(B)** test cohort.

As can be seen from Table 1, the optimal model was obtained by using the T2WI-DLR features combined with an SVM-based RFE feature selection method, and an SVM classifier. The AUC value of the model on the training and test cohort reached 0.86 (95% CI: 0.81, 0.91) and 0.85 (95% CI: 0.75, 0.95) for distinguishing brain abscess from cystic glioma, respectively. Figure 4 presents the performance of the optimal model. It can be seen from the figure that on the training set, the AUC values of the nested 5-fold were 0.86, 0.86, 0.92, 0.88, and 0.80, respectively. Besides, the standard deviation of the mean AUC value was 0.04, indicating that our model has good stability and robustness and reduces overfitting. The optimal cutoff value of the model was determined by Youden Index. On the test cohort, the sensitivity and specificity of the model were 73.1 and 87.1%, respectively, with an optimal critical value of 0.512 and a Jorden index of 0.601. The details of HCR and DTL feature selection and model construction are listed in Tables 2, 3, respectively. The selection of the hyperparameters for each model is listed in Supplementary Table 3. Moreover, the ROC curves for each model were compared, and the Delong test results are detailed in Supplementary Table 4. It can be seen from the table, that T2WI-DLR features (AUC, 0.85) are superior to T2WI-DTL (AUC, 0.71; *P* = 0.0058; DeLong test).

**Table 2:** HCR model construction.

**Classifier**	**Feature selection method**	**T1**			**T2**		
		**Optimal**	**Mean AUC**	**AUC**	**Optimal**	**Mean AUC**	**AUC**
		**feature number**	**(training cohort)**	**(test cohort)**	**feature number**	**(training cohort)**	**(test cohort)**
LR	RFE(LR)	10	0.75	0.77	10	0.84	0.81
	RFE(SVM)	10	0.74	0.76	10	0.83	0.81
	LASSO	5	0.72	0.75	11	0.81	0.80
SVM	RFE(LR)	10	0.72	0.75	10	0.84	0.80
	RFE(SVM)	10	0.75	0.71	10	0.84	0.80
	LASSO	5	0.72	0.75	11	0.82	0.79
KNN	RFE(LR)	10	0.70	0.75	10	0.75	0.88
	RFE(SVM)	10	0.71	0.78	10	0.74	0.81
	LASSO	5	0.71	0.75	11	0.79	0.82
RFC	RFE(LR)	10	0.69	0.75	10	0.76	0.83
	RFE(SVM)	10	0.73	0.72	10	0.75	0.80
	LASSO	5	0.71	0.72	11	0.74	0.86

**Table 3:** DTL model construction.

**Classifier**	**Feature selection method**	**T1**			**T2**		
		**Optimal**	**Mean AUC**	**AUC**	**Optimal**	**Mean AUC**	**AUC**
		**feature number**	**(training cohort)**	**(test cohort)**	**feature number**	**(training cohort)**	**(test cohort)**
LR	RFE(LR)	10	0.84	0.72	10	0.87	0.73
	RFE(SVM)	10	0.89	0.80	10	0.87	0.68
	LASSO	0	–	–	9	0.81	0.75
SVM	RFE(LR)	10	0.86	0.70	10	0.86	0.85
	RFE(SVM)	10	0.90	0.71	10	0.83	0.69
	LASSO	0	–	–	9	0.81	0.75
KNN	RFE(LR)	10	0.72	0.54	10	0.82	0.75
	RFE(SVM)	10	0.73	0.60	10	0.81	0.61
	LASSO	0	–	–	9	0.80	0.63
RFC	RFE(LR)	10	0.69	0.71	10	0.79	0.69
	RFE(SVM)	10	0.72	0.69	10	0.82	0.74
	LASSO	0	–	–	9	0.72	0.71

### DTL Feature Visualization

**Figure 5:**
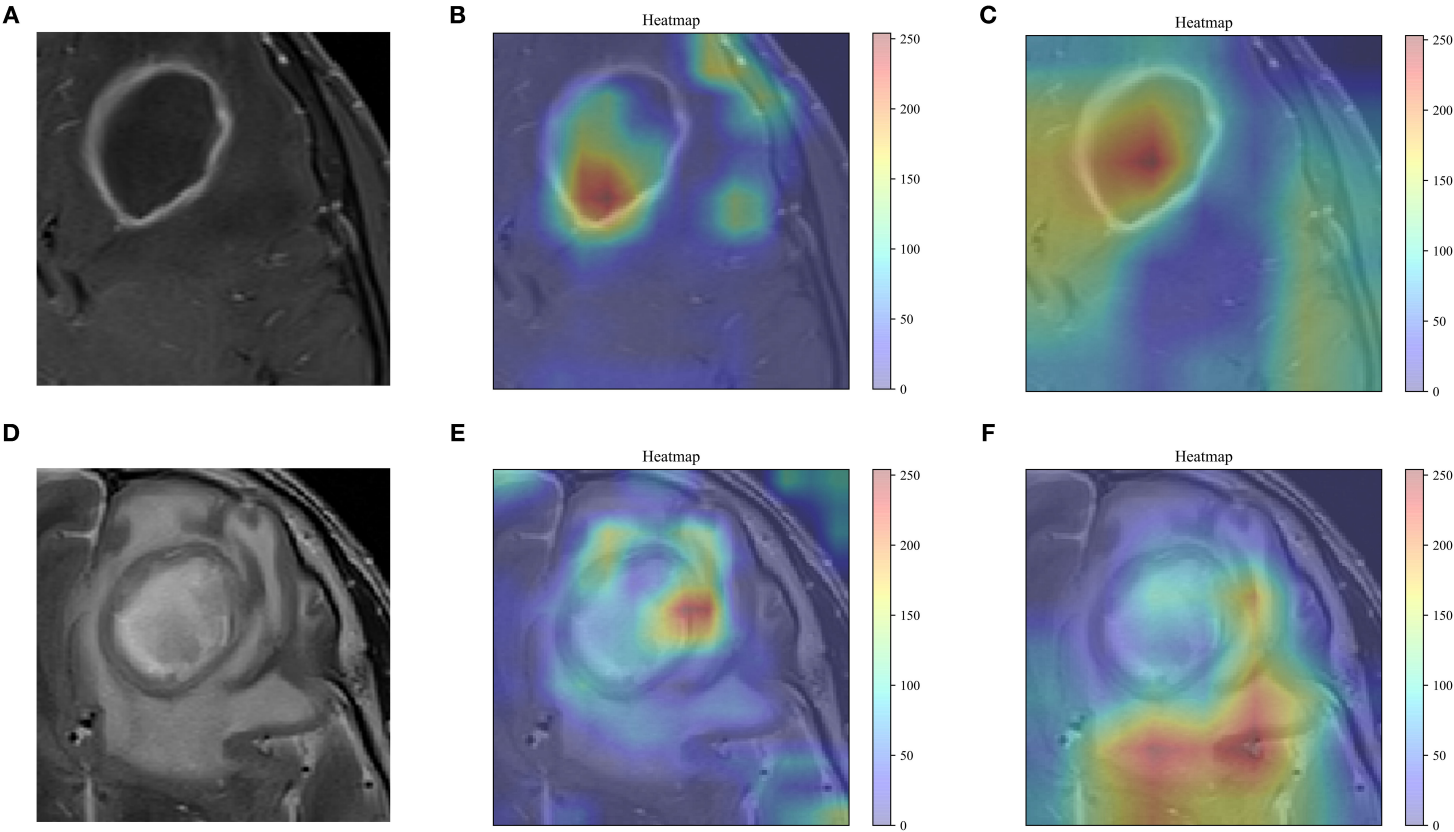
Feature visualization. **(A,D)** The grayscale T1WI, T2WI, and the corresponding heat map are shown, and the red areas indicating greater weighting, with color bars on the right side of the plot. **(B,E)**, the feature map of the last convolutional layer in the VGG-19 model. **(C,F)**, the feature map of the last convolutional layer in the Resnet-50 model.

As shown in Figure 5, the feature maps output by the last convolutional layer in the VGG-19 and Resnet-50 model are visualized. The feature map of the visually perceptible tumor region captures most of the details in the image. To a certain extent, it confirms the reliability of transfer learning for feature extraction. By visualizing the learning of the features in the image by these two networks, more insight into the working of the networks can be obtained, and the reasons why the disease may be correctly identified by transfer learning models can be understood.

### Clinical ADC Maps vs. Our Model

The cases containing ADC maps in the cohort were counted. There were 33 cases in total, including 21 cases of brain abscess and 12 cases of cystic glioma. A detailed comparison of the distinguishment performance between the clinical ADC maps diagnosis and our model is shown in Table 4. For each patient, the diagnosis of the radiologist and the model prediction are listed in Supplementary Table 5. It can be found that our model has the same accuracy as the clinical ADC assessment, so it has great potential for distinguishing between the two diseases.

**Table 4:** Clinical ADC maps vs. our model.

	**Accuracy**	**Precision**	**Recall**	**F1-score**	**Specificity**
ADC maps	0.848	0.818	0.75	0.783	0.905
T2WI-DLR	0.848	0.706	1	0.889	0.762

## Discussions

Accurate identification of brain abscesses and cystic gliomas is essential to planning appropriate treatment, assessing outcome, and future prognosis. However, due to the similarity in the conventional MR images, i.e., ring enhancement, it is difficult to distinguish between the two diseases. In this study, a deep learning-based statistical analysis method based on multistep feature selection and fusion was demonstrated and verified. The experimental results indicate that the method can be used to distinguish between brain abscess and cystic glioma in conventional T1WI and T2WI. The previous literature on disease prediction prognosis and classification differential diagnosis for quantitative image analysis has shown that deep learning contributes to better performance of radiomics analysis (21, 27, 31, 32). Our study demonstrates that by extracting DTL features with VGG-19, a model with excellent feature learning and feature representation abilities can be obtained. Besides, as shown in Figure 5, VGG-19 can better focus on the details of the tumor region than Resnet-50.

**Figure 6:**
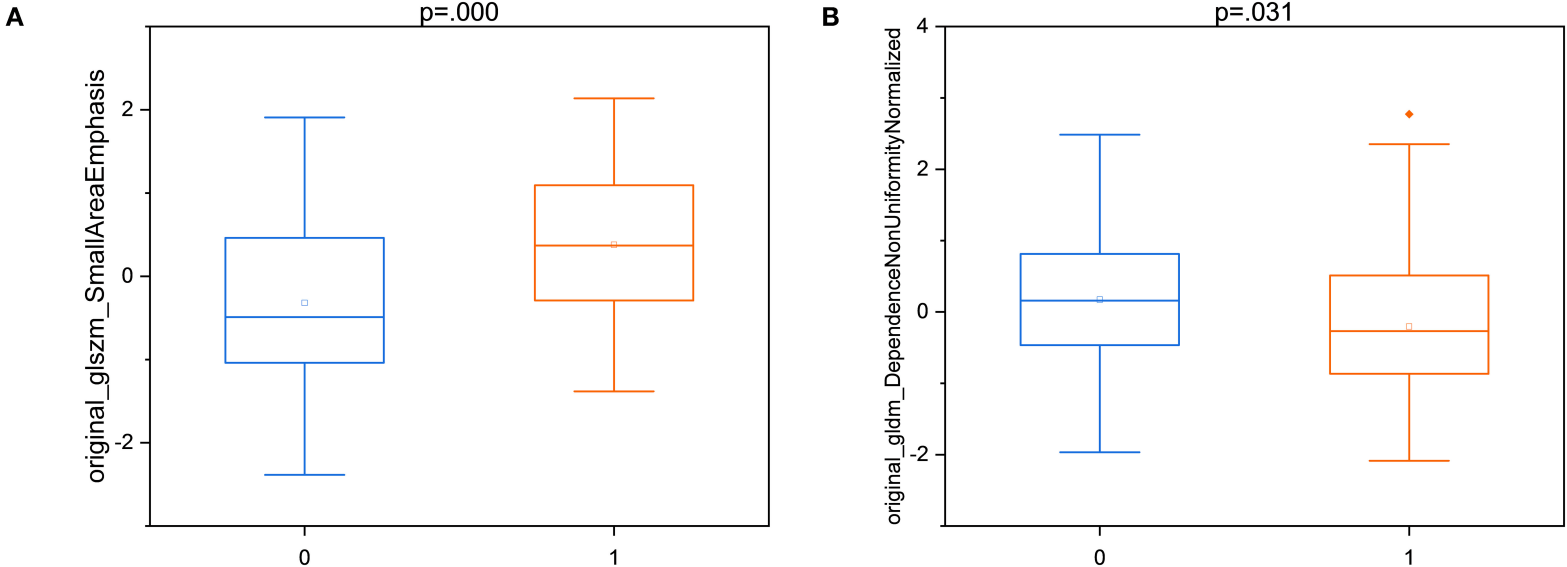
The box plots of two “good” T2WI-HCR of T2WI-DLR with the results of the statistical test. **(A)** original_glszm_SmallAreaEmphasis; **(B)** original_gldm_DependenceNonUniformityNormalized. Brain abscess:0; Cystic glioma:1.

According to the feature selection results of the optimal model, two “good” HCR features were selected for statistical significance analysis. It can be seen from the box plots in Figure 6 that there is not much difference in the distribution of the features between brain abscess and cystic glioma. All corresponding *p*-values of the statistical tests for distinguishing the two diseases are presented in the figure. These results indicate that these two features have a good identification ability in this work, showing the reproducibility and usefulness of feature engineering. Besides, the two “good” HCR features are all texture features, which reflect the homogeneous phenomenon in the image, which once again demonstrates the superiority of texture features in distinguishing brain abscess from cystic glioma. Previous studies have also shown that texture features are highly predictive in many tasks, which is consistent with the results obtained in this study (33, 34).

Compared with using T1WI and T2WI alone in DLR, the model based on combined modality does not achieve improved performance. This indicates that the single modality of T2WI is also a good predictor for distinguishing between brain abscesses and gliomas, and this is consistent with the fact that the T2 modality is more commonly used in imaging diagnosis of brain diseases in clinical practice. When the performance of the two models is stable and the results are complementary, model fusion can lead to better performance (35).

Previous studies (5, 36–38) have demonstrated that the advanced MRI techniques, such as magnetic resonance spectroscopy, susceptibility-weighted imaging, ADC, and dynamic susceptibility contrast-enhanced, can distinguish brain abscesses from gliomas, but these techniques have some limitations. Refer Supplementary Table 6 for model performance comparison. First, the sample size of these techniques is small, and only a few cases of pyogenic abscess and glioblastoma are included, hindering the direct application of the results to daily clinical practice. Second, the model based on the combination of intralesional susceptibility signal and ADC achieved a good AUC value (38). However, the combination does not lead to obvious improvement of differential diagnosis, because only a small number of patients with abscess/glioblastoma show atypical high/low ADC. Finally, none of the techniques were based on conventional MR images, increasing the image acquisition difficulty and cost. In this study, some measures were taken to overcome these limitations. The relatively larger sample size contributes to a better performance of conventional MR images for distinguishing brain abscesses and gliomas. The comparison of dataset size is listed in Supplementary Table 6. Also, the HCR and DTL features were extracted from conventional MR images. To our knowledge, there is no report on integrating HCR and DTL features for distinguishing brain abscesses from cystic gliomas. Besides, our research is based on some ordinary image data and does not require special training, so it has significant potential. In addition, DTL feature extraction uses a fixed-size bounding box for the tumor region, which not only provides information about intertumoral heterogeneity but also provides tumor microenvironment information to a certain extent.

To promote the development of radiomics as imaging biomarkers, a plethora of studies have used radiomics quality score (RQS) to evaluate and standardize radiomics (8, 39, 40). The RQS of our study was satisfactory at 15 points (41.7% of the ideal quality score), and the detailed result is listed in Supplementary Table 7. The RQSs of the relevant work (16–20) were analyzed in our study, but only our study is open to science and data, only one study conducted a multivariable analysis with non-radiomics features (20), and only one study based on multicenter validation (16). Besides, no research has conducted a phantom study, collected images of individuals at additional time points, discussed biological correlates, conducted a prospective study, or reported the cost-effectiveness of the clinical application.

The limitations of our work are as follows. First, due to the difficulty of obtaining external validation data, the patients in our study were single-center. The effects of clinical ADC maps diagnosis were compared with our proposed model, but this sequence was not added to our model due to the insufficient sample size of ADC maps. Multicenter validation, multi-MRI sequences, and prospective studies will be involved in our future work. Meanwhile, additional features such as proteomics, transcriptomics, pathomics, and genomic features were not considered in our study. Multi-omics joint analysis that integrates complex structural systems with multiple layers, levels, and functions may enhance the performance to identify brain abscess and cystic glioma and overcome the limitations of a single theoretical model. Besides, this study only used the image of the largest cross-section area with the upper and lower layers as the input to the VGG-19 or ResNet-50 model. The use of the 3D volume of the tumor/region of interest should be investigated in future research. Finally, the application of our study to identify other brain tumors and the enhancement of the algorithm will be explored.

## Conclusions

This paper first reports a model combining DTL features and HCR features from conventional MRI for distinguishing brain abscesses from cystic glioma. The study results provide an effective, inexpensive, convenient, and non-invasive method for differential diagnosis.

## Data Availability Statement

The original contributions presented in the study are included in the article/Supplementary Material, the main code are available from https://github.com/better0123/radiomics-analysis.git. Further inquiries can be directed to the corresponding author/s.

## Ethics Statement

The studies involving human participants were reviewed and approved by Institutional Review Board (IRB) of Xiangya Hospital. The patients/participants provided their written informed consent to participate in this study.

## Author Contributions

LB, ZZ, and ZJ: study design. ZL, QC, ZZ, XT, YW, and TC: data collection. LB, PH, ZZ, and CY: data analysis. DL, GY, and ZL: supervision. LB, QC, ZZ, and ZJ: manuscript writing. All authors contributed to the article and approved the submitted version.

## Funding

This study was supported by the National Nature Science Foundation of China (NO. 82073893, NO. 81873635, NO. 81703622 and NO. 81472693); the China Postdoctoral Science Foundation (NO. 2018M63302); the Natural Science Foundation of Hunan Province (NO. 2018JJ3838 and NO. 2018SK2101); the Hunan Provincial Health and Health Committee Foundation of China (C2019186); Xiangya Hospital Central South University postdoctoral foundation; the National Natural Science Foundation of China (61971271); the Taishan Scholars Project of Shandong Province (Tsqn20161023) and the Primary Research; Development Plan of Shandong Province (No. 2018GGX101018 and No. 2019QYTPY020).

## Conflict of Interest

The authors declare that the research was conducted in the absence of any commercial or financial relationships that could be construed as a potential conflict of interest.

## Publisher's Note

All claims expressed in this article are solely those of the authors and do not necessarily represent those of their affiliated organizations, or those of the publisher, the editors and the reviewers. Any product that may be evaluated in this article, or claim that may be made by its manufacturer, is not guaranteed or endorsed by the publisher.

## Abbreviations

DTL: deep transfer learning
HCR: hand-crafted radiomics
T1WI: T1-weighted image
T2WI: T2-weighted image
KNN: k-nearest neighbors
RFC: random forest classifier
LR: logistic regression
SVM: support vector machine
AUC: Area Under the Curve
DLR: deep learning-based radiomics
ADC: apparent diffusion coefficients
CNN: convolutional neural networks
LASSO: least absolute shrinkage and selection operator
RFE: recursive feature elimination
comb-HCR: combined HCR
comb-DTL: combined DTL
comb-DLR: combined DLR
ROC: receiver operating characteristic
RQS: radiomics quality score.
