# Supplementary material for: Differentiation of Brain Abscess From Cystic Glioma Using Conventional MRI Based on Deep Transfer Learning Features and Hand-Crafted Radiomics Features
Source: Front Med (Lausanne). 2021 Nov 12;8:748144. doi: 10.3389/fmed.2021.748144 (PMC8636043; doi:10.3389/fmed.2021.748144)
Supplement: Supplementary file 1 [file Table_1.DOCX]

Supplementary Material

# Supplementary Figures and Tables

## Supplementary Tables

**Supplementary Table 1.** Baseline characteristics

| Characteristic | Training cohort(N=131) | |  | Test cohort(N=51) | |  |
| --- | --- | --- | --- | --- | --- | --- |
|  | Brain Abscess(+)(N=71) | Cystic Glioma(-)(N=60) | P | Brain Abscess(+)(N=31) | Cystic Glioma(-)(N=26) | P |
| Age,mean+-SD,YEARS | 46.5634+-16.4766 | 45.7667+-15.3229 | 0.5465 | 50.5484+-19.9730 | 47.0769+-14.9424 | 0.3279 |
| Gender,N.(%) | 0.5782 | | | 0.5014 | | |
| Male | 27(30.0%) | 20(33.3%) |  | 6(19.4%) | 7(26.9%) |  |
| Female | 44(70.0%) | 40(66.7%) |  | 25(80.6%) | 19(73.1%) |  |

**Supplementary Table 2.** The detailed feature selection results.

|  | HCR | DTL | DLR |
| --- | --- | --- | --- |
| T1 | original_ngtdm_Strength | 370 | original_glcm_MaximumProbability |
|  | original_glcm_MaximumProbability | 11 | 370 |
|  | original_firstorder_Maximum | 66 | 11 |
|  | original_firstorder_Minimum | 435 | 66 |
|  | original_shape_VoxelVolume | 345 | 435 |
|  | original_firstorder_Range | 449 | 345 |
|  | original_glszm_GrayLevelNonUniformity | 108 | 449 |
|  | original_glcm_ClusterProminence | 13 | 108 |
|  | original_glszm_SmallAreaEmphasis | 208 | 13 |
|  | original_firstorder_Mean | 458 | 208 |
| T2 | original_glszm_SmallAreaEmphasis | 500 | original_glszm_SmallAreaEmphasis |
|  | original_firstorder_Skewness | 45 | original_gldm_DependenceNonUniformityNormalized |
|  | original_glcm_Correlation | 295 | 500 |
|  | original_ngtdm_Contrast | 77 | 45 |
|  | original_gldm_SmallDependenceHighGrayLevelEmphasis | 204 | 295 |
|  | original_glrlm_RunEntropy | 8 | 8 |
|  | original_glszm_SmallAreaHighGrayLevelEmphasis | 106 | 106 |
|  | original_glszm_GrayLevelVariance | 186 | 186 |
|  | original_gldm_DependenceNonUniformityNormalized | 55 | 55 |
|  | original_firstorder_Range | 86 | 86 |
| Combined modality | original_ngtdm_StrengthT1WI | 370T1WI | original_glcm_MaximumProbabilityT1WI |
|  | original_glcm_MaximumProbabilityT1WI | 11T1WI | 370T1WI |
|  | original_glszm_GrayLevelNonUniformityT1WI | 66T1WI | 11T1WI |
|  | original_glszm_SmallAreaEmphasisT2WI | 345T1WI | 345T1WI |
|  | original_firstorder_SkewnessT2WI | 108T1WI | 449T1WI |
|  | original_ngtdm_ContrastT2WI | 500T1WI | 208T1WI |
|  | original_glrlm_RunEntropyT2WI | 295T2WI | original_glszm_SmallAreaEmphasisT2WI |
|  | original_glszm_SmallAreaHighGrayLevelEmphasisT2WI | 8T2WI | 186T2WI |
|  | original_gldm_DependenceNonUniformityNormalizedT2WI | 106T2WI | 55T2WI |
|  | original_firstorder_RangeT2WI | 86T2WI | 86T2WI |

**Supplementary Table 3.** Hyperparameters selection of each model.

| Hyperparameters selection | | SVC | | | LR | | | |
| --- | --- | --- | --- | --- | --- | --- | --- | --- |
|  |  | C(1, 30, 1) | Kernal("linear") | gamma(-5, 1, 1000, base=10) | C(1, 30, 1) | Penalty(L1;L2) | Solver(L1:"liblinear", "saga";L2:"sag", "newton-cg", "lbfgs", "liblinear", "saga") | Max_iter |
| HCR | T1 WI-HCR | - | - | - | 1 | L2 | sag | 10000 |
|  | T2WI-HCR | 19 | linear | 1.00E-05 | - | - | - | - |
|  | comb-HCR | - | - | - | 5 | L2 | sag | 10000 |
| DTL | T1 WI-DTL | 5 | linear | 1.00E-05 | - | - | - | - |
|  | T2WI-DTL | 3 | linear | 1.00E-05 | - | - | - | - |
|  | comb-DTL | - | - | - | 1 | L2 | sag | 10000 |
| DLR | T1 WI-DLR | - | - | - | 8 | L1 | liblinear | 10000 |
|  | T2 WI-DLR | 5 | linear | 1.00E-05 | - | - | - | - |
|  | comb-DLR | 5 | linear | 1.00E-05 | - | - | - | - |

**Supplementary Table 4.** Delong test.

| Delong test | T1 WI-HCR | T2WI-HCR | comb-HCR | T1 WI-DTL | T2WI-DTL | comb-DTL | T1 WI-DLR | T2 WI-DLR | comb-DLR |
| --- | --- | --- | --- | --- | --- | --- | --- | --- | --- |
| T1 WI-HCR | - | 0.5303 | 0.9229 | 0.7946 | 0.3515 | 0.8239 | 0.5406 | 0.4312 | 0.5279 |
| T2WI-HCR | - | - | 0.4751 | 0.431 | 0.7112 | 0.7454 | 0.9119 | 0.1517 | 0.1832 |
| comb-HCR | - | - | - | 0.764 | 0.3679 | 0.8714 | 0.6273 | 0.3341 | 0.4347 |
| T1 WI-DTL | - | - | - | - | 0.2154 | 0.5767 | 0.0746 | 0.5823 | 0.6877 |
| T2WI-DTL | - | - | - | - | - | 0.3452 | 0.6158 | **0.0058** | 0.0748 |
| comb-DTL | - | - | - | - | - | - | 0.7481 | 0.2025 | 0.364 |
| T1 WI-DLR | - | - | - | - | - | - | - | 0.1784 | 0.1476 |
| T2 WI-DLR | - | - | - | - | - | - | - | - | 0.7713 |
| comb-DLR | - | - | - | - | - | - | - | - | - |

**Supplementary Table 5.**  Clinical ADC maps versus our model. Brain abscess:0; Cystic glioma:1.

|  | Actual | ADC maps | T2WI-DLR |
| --- | --- | --- | --- |
| 1 | 1 | 1 | 0 |
| 2 | 1 | 1 | 1 |
| 3 | 0 | 0 | 0 |
| 4 | 1 | 0 | 0 |
| 5 | 0 | 0 | 0 |
| 6 | 0 | 0 | 0 |
| 7 | 0 | 0 | 0 |
| 8 | 0 | 0 | 0 |
| 9 | 0 | 0 | 0 |
| 10 | 0 | 0 | 0 |
| 11 | 1 | 0 | 0 |
| 12 | 1 | 1 | 1 |
| 13 | 0 | 0 | 0 |
| 14 | 0 | 0 | 0 |
| 15 | 0 | 0 | 0 |
| 16 | 0 | 0 | 0 |
| 17 | 1 | 1 | 1 |
| 18 | 1 | 1 | 1 |
| 19 | 1 | 1 | 0 |
| 20 | 1 | 1 | 1 |
| 21 | 0 | 0 | 0 |
| 22 | 0 | 0 | 0 |
| 23 | 0 | 0 | 0 |
| 24 | 0 | 0 | 0 |
| 25 | 0 | 0 | 0 |
| 26 | 0 | 0 | 0 |
| 27 | 1 | 0 | 0 |
| 28 | 1 | 1 | 1 |
| 29 | 1 | 1 | 1 |
| 30 | 0 | 0 | 0 |
| 31 | 0 | 0 | 0 |
| 32 | 0 | 0 | 0 |
| 33 | 0 | 1 | 0 |

**Supplementary Table 6.**  Dataset size and model performance comparison.

|  | dataset(N) | AUC | Accuracy(%) | Sensitivity(%) | Specificity(%) |
| --- | --- | --- | --- | --- | --- |
| Toh, Cheng Hong, et al. | 56 | 0.85 | 78 | 74 | 82 |
| Fu, Jui-Hsun, et al. | 44 | 0.91 | 88 | 86 | 91 |
| Lai, Ping-Hong, et al. | 78 | 1 | 100 | 100 | 100 |
| This research | 188 | 0.85 | 77 | 76 | 74 |

**Supplementary Table 7.**  The radiomics quality score.

| Criteria | Total Points | Points |
| --- | --- | --- |
| Image protocol quality | +1 or +2 | +1 |
| Multiple segmentation | +1 | +1 |
| Phantom study | +1 | +0 |
| Imaging at multiple time points | +1 | +0 |
| Feature reduction or adjustment for multiple testing | -3 or +3 | +3 |
| Multivariable analysis | +1 | +0 |
| Biological correlates | +1 | +0 |
| Cut-oﬀ analysis | +1 | +1 |
| Discrimination statistics | +1 or +2 | +2 |
| Calibration statistics | +1 or +2 | +0 |
| Prospective study | +7 | +0 |
| Validation | -5 to +5 | +2 |
| Comparison to ‘gold standard’ | +2 | +2 |
| Potential clinical applications | +2 | +0 |
| Cost-eﬀectiveness analysis | +1 | +0 |
| Open science and data | +1 to +4 | +3 |
| RQS Total | 36 | 15 |
